# Differences in Gut Metabolites and Microbial Composition and Functions between Egyptian and U.S. Children Are Consistent with Their Diets

**DOI:** 10.1128/mSystems.00169-16

**Published:** 2017-02-07

**Authors:** V. Shankar, M. Gouda, J. Moncivaiz, A. Gordon, N. V. Reo, L. Hussein, O. Paliy

**Affiliations:** a Department of Biochemistry and Molecular Biology, Boonshoft School of Medicine, Wright State University, Dayton, Ohio, USA; b Department of Human Nutrition, National Research Centre, Giza, Egypt; Vall d'Hebron Research Institute

**Keywords:** children, diet, metabolomics, metagenomics, microbiota, nutrition

## Abstract

Previous studies indicated that populations consuming a Mediterranean diet rich in fiber, vegetables, and fruits have a significantly lower risk of cardiovascular and metabolic diseases than populations of industrialized societies consuming diets enriched in processed carbohydrates, animal proteins, and fats. To explore the potential contributions of gut microbiota to the observed diet-related metabolic effects, we conducted an integrative analysis of distal gut microbiota composition and functions and intestinal metabolites in Egyptian and U.S. teenagers. All Egyptian gut microbial communities belonged to the *Prevotella* enterotype, whereas all but one of the U.S. samples were of the *Bacteroides* enterotype. The intestinal environment of Egyptians was characterized by higher levels of short-chain fatty acids, a higher prevalence of microbial polysaccharide degradation-encoding genes, and a higher proportion of several polysaccharide-degrading genera. Egyptian gut microbiota also appeared to be under heavier bacteriophage pressure. In contrast, the gut environment of U.S. children was rich in amino acids and lipid metabolism-associated compounds; contained more microbial genes encoding protein degradation, vitamin biosynthesis, and iron acquisition pathways; and was enriched in several protein- and starch-degrading genera. Levels of 1-methylhistamine, a biomarker of allergic response, were elevated in U.S. guts, as were the abundances of members of *Faecalibacterium* and *Akkermansia*, two genera with recognized anti-inflammatory effects. The revealed corroborating differences in fecal microbiota structure and functions and metabolite profiles between Egyptian and U.S. teenagers are consistent with the nutrient variation between Mediterranean and Western diets.

**IMPORTANCE** The human gastrointestinal microbiota functions as an important mediator of diet for host metabolism. To evaluate how consumed diets influence the gut environment, we carried out simultaneous interrogations of distal gut microbiota and metabolites in samples from healthy children in Egypt and the United States. While Egyptian children consumed a Mediterranean diet rich in plant foods, U.S. children consumed a Western diet high in animal protein, fats, and highly processed carbohydrates. Consistent with the consumed diets, Egyptian gut samples were enriched in polysaccharide-degrading microbes and end products of polysaccharide fermentation and U.S. gut samples were enriched in proteolytic microbes and end products of protein and fat metabolism. Thus, the intestinal microbiota might be selected on the basis of the diets that we consume, which can open opportunities to affect gut health through modulation of gut microbiota with dietary supplementations.

## Abstract (precis)

The human gastrointestinal microbiota functions as an important mediator of diet for host metabolism. To evaluate how consumed diets influence the gut environment, we carried out simultaneous interrogations of distal gut microbiota and metabolites in samples from healthy children in Egypt and the United States. While Egyptian children consumed a Mediterranean diet rich in plant foods, U.S. children consumed a Western diet high in animal protein, fats, and highly processed carbohydrates. Consistent with the consumed diets, Egyptian gut samples were enriched in polysaccharide-degrading microbes and end products of polysaccharide fermentation, and U.S. gut samples were enriched in proteolytic microbes and end products of protein and fat metabolism. Thus, the intestinal microbiota might be selected on the basis of the diets that we consume, which can open opportunities to affect gut health through modulation of gut microbiota with dietary supplementations.

## INTRODUCTION

Throughout human evolution, dramatic shifts in the lifestyle and geographical distribution of the human species have led to periodic changes in consumed diets and nutritional intakes. When the original human societies were primarily hunter-gatherers, their diet was rich in lean wild animal meat and seafood as well as in what we now call dietary fiber from roots, tubers, fruits, and leafy vegetables (1, 2). The advent of agriculture about 12,000 years ago and the accompanying change from a nomadic to a sedentary lifestyle (3) led to a dramatic shift toward the consumption of large quantities of refined grains, seeds, and, eventually, simple sugars such as sucrose and fructose (2, 4). Over time, animal husbandry also developed and became an economically viable source of food, such that the diet of the modern industrialized societies now contains significant amounts of animal proteins and fats.

These shifts in dietary habits in different geographical regions have historically given rise to several different diet types. The typical “Western” diet consumed by the majority of populations in most industrialized countries is rich in animal proteins and fats, dairy products, and refined, starch-enriched grains, cereals, flour, and sugars (4–6). Modern agricultural and husbandry methods coupled with the ability to preserve perishable foods made these products profitable to produce on a large scale. The level of consumption of fruits, nuts, and vegetables in the Western diet is generally low, and intake of dietary fiber is well below recommended levels (7, 8). This diet, enriched in refined carbohydrates, animal fat, and protein, has been postulated to be one of the primary causes for the rising number of metabolic diseases in the industrialized countries (4, 9). In contrast, the “Mediterranean” diet is considered the standard of healthy nutrition and has been shown to be associated with an increased life span and a low incidence of metabolic and cardiovascular diseases (10, 11). Consumed by populations of the Mediterranean Sea region, it is high in fruits, vegetables, whole grain, beans, nuts, and plant fats, with a low fraction of meats and sweets (12, 13).

In addition to the well-recognized direct effects of consumed nutrients on human physiology, diet is also considered to be one of the main determinants of gut microbiota composition and diversity (14, 15). Switching from a diet rich in animal products to one high in fiber and plant foods rapidly changes gut microbiota in humans and animals (16, 17). Reciprocally, the gut microbiota plays a large and vital role in the biotransformation of consumed foods. A significant proportion of ingested foods escapes digestion and absorption in the small intestine and reaches the colon. These include dietary fiber (nonstarch polysaccharides), resistant starch, small amounts of simpler carbohydrates, and some proteins and fats, as well as bile acids and enzymes released in the small intestine (18, 19). Most of these unabsorbed compounds are fermented in the colon by gut microbes. The end products of microbial metabolism have been shown to have many positive (short-chain fatty acids [SCFAs]) as well as negative (trimethylamine, ammonia, hydrogen sulfide) effects on the host health (19, 20). Alterations of the gut microbial populations have been associated with the development of metabolic disorders such as obesity and type 2 diabetes (21, 22), and gut microbiota changes during childhood can have lifelong effects (23, 24).

In this study, we aimed to discover possible relationships between human gut microbiota and consumed diets. To achieve that goal, we compared fecal microbiota structures and functions as well as fecal metabolites in two cohorts of children: teenagers from the United States consuming a typical Western diet and population group age-matched Egyptians consuming a Mediterranean-type diet. Simultaneous analyses of microbial community membership and functional gene pool data combined with the quantification of metabolites in the same samples allowed integrative analysis of these data sets and revealed links between the gut microbiota and the intestinal environment.

## RESULTS

### Gut microbiota compositions differ between healthy U.S. and Egyptian teenagers.

**FIG 1:**
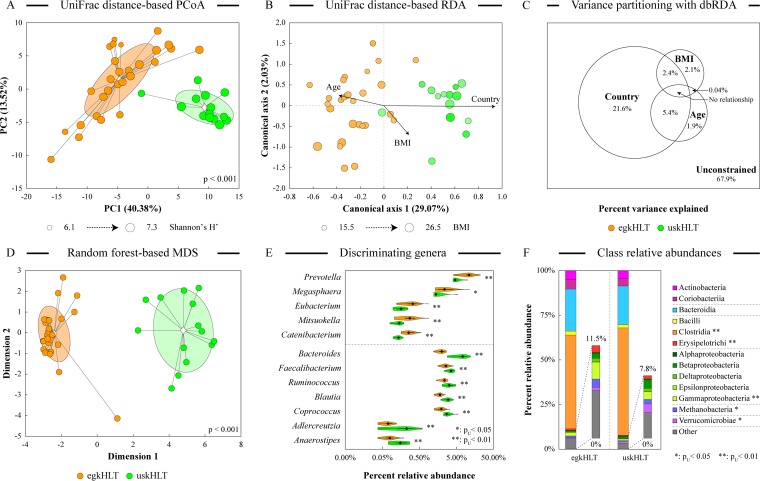
Comparison of distal gut microbiota composition between Egyptian and U.S. groups. (A and B) Sample similarity was assessed by unconstrained weighted UniFrac-based principal-coordinate analysis (A) and constrained weighted UniFrac-based redundancy analysis (B) run on the chord-transformed genus abundance data set. The statistical significance of group separation in PCoA is based on the Davies-Bouldin index. Group clouds represent areas of three standard errors around the group centroid (diamond), and dot sizes in PCoA are proportional to Shannon’s H′ alpha diversity values for that sample. Distance-based RDA used three explanatory variables (group, age, and body mass index). The age and the BMI of the subjects are represented by color gradients and the size of each dot, respectively. Arrows in the db-RDA biplot denote the magnitudes and directions of the effects of explanatory variables. (C) A variation partitioning diagram depicts the relative contributions of explanatory variables to the overall variability in the data set. (D) Results of random forest discriminant analysis of chord-transformed genus abundances were visualized through multidimensional scaling of the sample proximity matrix. The statistical significance of group separation is based on the Davies-Bouldin index. Group clouds represent areas of three standard errors around the group centroid (diamond). MDS, multidimensional scaling. (E) The relative abundances of the top 12 RF discriminatory genera are depicted on a violin plot. Each violin shows the density distribution of genus abundances among all samples in the group. (F) Community structure at the class level. Classes are ordered according to the phylum. Where shown, single asterisks (*) and double asterisks (**) indicate statistical significance of taxon abundance differences between two groups (p_u_ < 0.05 and p_u_ < 0.01, respectively) based on the FDR-adjusted Mann-Whitney *U* test.

High-throughput 16S rRNA gene amplicon sequencing was used to analyze microbial composition in fresh fecal samples collected from 28 Egyptian teenagers and 14 age-matched teenagers from the Midwest region of United States. Exploratory principal-coordinate analysis (PCoA) using phylogenetically defined weighted UniFrac distance measure (25) distributed samples in the ordination space based largely on their group identity. Figure 1A shows PCoA results based on the analysis of the genus abundance data set; Fig. S1 in the supplemental material displays PCoA ordination results for the phylotype data set. We also calculated Bray-Curtis (BC) beta diversity distances among all samples. Consistent with the PCoA findings, average intersample BC distances between samples from healthy preadolescent and adolescent male volunteers from Giza, Egypt (designated egkHLT), and from Dayton, OH (designated uskHLT), were significantly larger than the intragroup distances (0.640 versus 0.569 and 0.576; *p*_*U*_ < 0.001). Using sample group, age, and body mass index (BMI) values as explanatory variables, we then conducted constrained ordination analysis using distance-based redundancy analysis (db-RDA) and a weighted UniFrac distance matrix (Fig. 1B). The three explanatory variables together explained 32.1% of the overall variability in the microbiota composition. Variation partitioning and sample dispersal in the constrained ordination space indicated that sample group assignment was the dominant gradient in the data set, accounting for 29.3% of the overall variability. In contrast, age and BMI displayed smaller effects (Fig. 1C). A large portion of the overall microbiota composition variation was not accounted for by the available explanatory variables. This finding is consistent with the previously observed high interpersonal variability in human gut microbiota composition (26, 27).

10.1128/mSystems.00169-16.1 FIG S1  Comparison of distal gut microbiota compositions between Egyptian and U.S. groups using OTU abundances. Relationships between samples were assessed using weighted UniFrac-based principal-coordinate analysis performed on chord-transformed OTU abundance data sets. Statistical significance of group separation in PCoA is based on the Davies-Bouldin index. Group clouds represent areas of three standard errors around the group centroid (diamond). Download FIG S1, TIF file, 0.3 MB. Copyright © 2017 Shankar et al. 2017 Shankar et al. This content is distributed under the terms of the Creative Commons Attribution 4.0 International license.

### Specific microbial genera are differentially abundant between Egyptian and U.S. teenagers.

To identify specific genera that contributed to the observed separation of samples between groups in ordination analyses, random forest (RF) discriminant analysis was performed on the genus abundance data set. The RF model clearly separated the two groups of samples, as shown in Fig. 1D, and variable importance scores (calculated as the increase in model error due to the permutation of the variable) were used to define the major discriminatory genera (see Table S4 in the supplemental material) (28). Figure 1E displays the distribution of abundances of the top 12 discriminating genera among egkHLT and uskHLT samples; a list of all genus abundances together with the *P* values of Mann-Whitney *U* test for significant differences between groups is provided in Table S1. While many differences were observed at the genus level, such distinctions between groups were less pronounced at the class level. Levels of *Gammaproteobacteria* and *Methanobacteria* were statistically significantly higher in egkHLT, while levels of *Clostridia* and *Verrucomicrobia* were higher in uskHLT (see Fig. 1F and Table S2). Considering the differentially abundant genera, the higher abundance of *Prevotella* in egkHLT samples and the reciprocal higher abundance of *Bacteroides* in the uskHLT samples (both are members of class *Bacteroidia*) are consistent with several previous studies that indicated a higher prevalence of members of *Bacteroides* in samples from the United States, western Europe, and industrialized Asian countries than in samples from less-industrialized and more-rural populations (29–33). Many *Bacteroides* members can utilize proteins for growth (34, 35), which might explain their prevalence in subjects from developed countries consuming typical Western diets heavy in animal fats and proteins. On the other hand, *Prevotella* spp. are known degraders of xylan and other fibrous polysaccharides (36), which is consistent with their presence in ethnic groups where a large fraction of the diet is comprised of vegetables and grains (29, 30). Other genera enriched in the adolescent Egyptian gut included polysaccharide-degrading *Megasphaera*, *Eubacterium*, *Mitsuokella*, and *Catenibacterium* (37–39). *Catenibacterium* and *Mitsuokella* were also found in the stool of Bangladeshi children but not in the samples from the cohort of U.S. kids (31), and both *Catenibacterium* and *Eubacterium* were previously linked to the abundances of *Prevotella* (26, 32). The gut microbiota of Egyptian children was also enriched in several genera typically associated with pathogenicity and infections, including *Succinivibrio* and *Treponema* (see Table S1) (29, 30). The presence of *Treponema* might relate to the ability of some members of this genus to degrade xylan and cellulose (29, 33).

10.1128/mSystems.00169-16.6 TABLE S1  Relative abundances of distal gut microbial genera from Egyptian and U.S. children. Download TABLE S1, XLSX file, 0.2 MB. Copyright © 2017 Shankar et al. 2017 Shankar et al. This content is distributed under the terms of the Creative Commons Attribution 4.0 International license.

10.1128/mSystems.00169-16.7 TABLE S2  Relative abundances of distal gut microbial classes from Egyptian and U.S. children. Download TABLE S2, XLSX file, 0.2 MB. Copyright © 2017 Shankar et al. 2017 Shankar et al. This content is distributed under the terms of the Creative Commons Attribution 4.0 International license.

In comparison, species of several known starch-degrading genera, namely, *Ruminococcus*, *Coprococcus*, and *Blautia* (40), were more abundant in the stools of U.S. children, possibly due to the high prevalence of starch as a dietary polysaccharide in the Western diet (41). In addition, abundances of *Bilophila*, a genus that is associated with bile acids and high-fat diets (42), mucin-degrading genus *Akkermansia*, and clostridial genus *Faecalibacterium* were 1.5-fold to 4-fold higher in the uskHLT samples (see Table S1). *Akkermansia* and *Faecalibacterium* were previously shown to exert anti-inflammatory effects on the intestinal mucosa and adipose tissues (43, 44). Considering the significantly higher incidence of autoimmune diseases in Western populations (45), the higher abundance of this genus in the gut of U.S. teenagers might indicate a host-driven adaptation to elevated inflammatory levels.

### Egyptian and U.S. gut microbiotas belong to different enterotypes.

**FIG 2:**
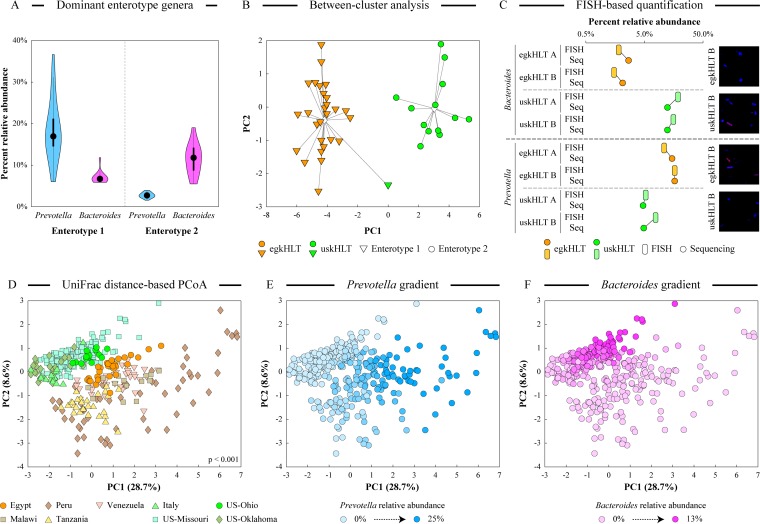
Enterotypes of the distal gut microbial profiles. (A) Relative abundances of the main enterotype drivers, *Prevotella* and *Bacteroides*, among the two identified clusters. Each violin shows the density distribution of genus abundance among all samples within the cluster (thickness of the violin), median value (black dot), and 25% to 75% range of values (black bar). (B) Sample clustering into different enterotypes based on the between-cluster ordination analysis. (C) Comparison of *Prevotella* and *Bacteroides* relative abundances in four samples based on sequencing (Seq) and fluorescent *in situ* hybridization (FISH) results. Representative fluorescent images are shown on the right; pink coloring corresponds to genus-specific fluorescent probes, and blue coloring represents DAPI DNA staining. (D) Results of weighted UniFrac distance-based PCoA performed using OTU abundances showing separation of fecal samples between industrialized and nonindustrialized countries. The statistical significance of group separation is based on the Davies-Bouldin index. Panels E and F display the same PCoA space, with relative abundances of *Prevotella* (E) and *Bacteroides* (F) overlaid as dot color gradients.

Following the recently described discovery of different enterotypes of human distal gut microbiota (26, 46, 47), we assessed if any such enterotypes could be revealed in the microbial communities profiled in the egkHLT and uskHLT samples. Calinski-Harabasz (CH) index values (Fig. S5) indicated that microbial data set can be optimally distributed into two enterotypes, and samples were separated into these enterotypes utilizing the partitioning around the medoid (PAM) clustering algorithm (48). With the exception of a single uskHLT sample which had an unusually large representation of *Prevotella* (15.8% compared with 7.7% average for uskHLT), all uskHLT samples clustered into the “*Bacteroides*” enterotype, and all egkHLT samples clustered into the “*Prevotella*” enterotype (see Fig. 2A and B). Previously, in a diet assessment study, the *Bacteroides* enterotype was strongly associated with protein and fat consumption, whereas the *Prevotella* enterotype was associated with carbohydrate consumption (26). These associations are consistent with the differences between the typical U.S. diet (Western diet; high in animal protein and fats) and the typical Egyptian diet (Mediterranean diet; high in plant polysaccharides). To confirm our sequencing results, we utilized fluorescent *in situ* hybridization (FISH) to visualize *Bacteroides* and *Prevotella* cells in select egkHLT and uskHLT samples. As shown in Fig. 2C, a good concordance between sequencing and FISH results was observed, lending additional support to the idea of the significance of the observed *Bacteroides*-*Prevotella* reciprocity between the uskHLT and egkHLT groups.

### Distal gut microbiotas separate subjects from Western and developing countries.

Extending our observation of the similarities of the gut microbiota differences between U.S. and Egyptian children to the results of several previous studies, we sought to carry out a cumulative ordination analysis of these data sets. Because it was shown previously that the choice of the interrogated 16S rRNA gene variable region has an impact on the estimates of microbial composition (49), we limited our comparison to studies that used the same V4 variable region of prokaryotic 16S rRNA gene to obtain a phylogenetic profile of gut microbiota. We combined our high-throughput amplicon sequencing data set (Illumina MiSeq platform) with a U.S.-versus-Malawi-versus-Venezuela subject comparison (Illumina HiSeq platform) (50), a Tanzania-versus-Italy subject comparison (Roche 454 FLX titanium platform) (30), and a U.S.-versus-Peru subject comparison (Illumina HiSeq platform) (33). UniFrac distance-based ordination PCoA of the combined data set revealed a statistically significant separation of fecal samples between industrialized countries (Italy and United States) and developing countries (Egypt, Malawi, Venezuela, Tanzania, and Peru) (Fig. 2D). Discriminatory random forest analysis and orthogonal projections to latent structures discriminant analysis (OPLS-DA) separated these two groups of samples well and designated *Prevotella* and *Bacteroides* the top separating genera (see Fig. S2; note that the random forest proximity matrix projection also separated our study samples from the others, which was likely due to technical variations among studies). The abundance gradients of these genera aligned along the primary axes of variability in the cumulative PCoA (Fig. 2E and F). Thus, we can speculate that there is a general dissimilarity of human distal gut microbiotas between industrialized populations and “developing” societies illustrated by the *Prevotella*-*Bacteroides* dichotomy.

10.1128/mSystems.00169-16.2 FIG S2  Discriminant analyses of distal gut microbial profiles from Western and developing countries. Results of random forest (RF) (A) and orthogonal projection to latent structures (OPLS-DA) (B) discriminant analyses performed on chord-transformed genus abundances show statistically significant separation of fecal samples between industrialized and nonindustrialized countries. Statistical significance of group separation on ordination plots is based on the Davies-Bouldin index. The top 10 discriminatory genera based on the mean decrease in accuracy of RF data and absolute values of genus weights in OPLS-DA are listed on the left of the respective plots. Download FIG S2, TIF file, 0.6 MB. Copyright © 2017 Shankar et al. 2017 Shankar et al. This content is distributed under the terms of the Creative Commons Attribution 4.0 International license.

### Differences in distal gut metabolites reflect dietary preferences.

**FIG 3:**
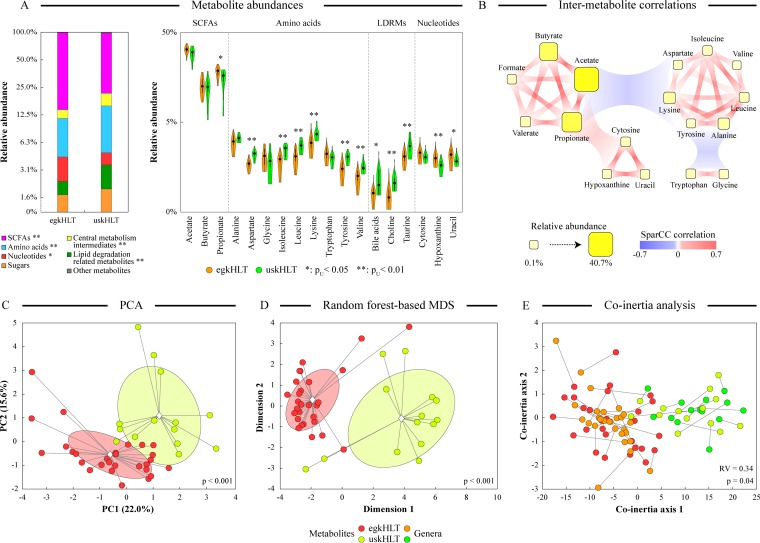
Comparison of distal gut metabolite profiles between Egyptian and U.S. groups. (A) Overall distribution of metabolite categories among egkHLT and uskHLT samples shown on a stacked-column graph. The relative abundances of individual measured metabolites are represented as violin plots. Where shown, single asterisks (*) and double asterisks (**) indicate statistical significance of taxon abundance differences between two groups (p_u_ < 0.05 and p_u_ < 0.01, respectively) based on the FDR-adjusted Mann-Whitney *U* test. LDRMs, lipid degradation-related metabolites. (B) Statistically significant SparCC-based correlations among individual metabolites and metabolite groups. The associations between metabolite categories represent the median of pairwise correlations among individual metabolites from different categories. (C and D) Sample distribution in ordination space based on the centered log-ratio transformed metabolite relative abundances from principal-component analysis (C) and random forest discriminant analysis (D). *P* values indicate statistical significance of separation of sample groups based on the Davies-Bouldin index. Group clouds represent three standard errors around the group centroids (diamond). MDS, multidimensional scaling. (E) Coinertia analysis showing congruency of sample dispersal in ordination space based on metabolite and genus abundance profiles. The distance between the positions of each sample on two ordination plots is indicated by a connecting line. Shorter lines represent similar sample positions in the plots. Statistical significance and the relative fit of the ordinations were assessed by *P* value and RV coefficient, respectively.

To assess whether the differences in microbiota composition and consumed diets between Egyptian and U.S. teenagers can alter luminal environment, we employed proton nuclear magnetic resonance (NMR) to obtain metabolite profiles from all collected stool samples. ^1^H NMR spectra were recorded for all water-soluble fecal extracts, and a dynamic binning algorithm was utilized to digitize spectral data (overlaid ^1^H NMR spectra are shown in Fig. S3). Exploratory principal-component analysis (PCA), as well as discriminatory RF analysis and ordination OPLS-DA, separated the samples with statistical significance according to the group assignment (see Fig. S4). A spectral deconvolution algorithm was then utilized to robustly quantify the levels of 32 metabolites in all interrogated samples. Statistically significant differences between egkHLT and uskHLT groups were observed for the fractional abundances of many metabolites (Fig. 3A and Table S3). The three major short-chain fatty acids (SCFAs), acetate, butyrate, and propionate, were the most abundant metabolites in every fecal sample and overall showed significantly higher levels in the gut of Egyptian teenagers. Because SCFAs are the end products of the fermentation of complex polysaccharides, the latter finding is consistent with the higher fraction of dietary fiber in Mediterranean diet (51). On the other hand, levels of seven of nine measured amino acids were higher in U.S. children (see Fig. 3A), consistent with the higher protein consumption in subjects consuming a Western diet (4, 52). One of the largest differences was observed for lysine, likely because the levels of this indispensable amino acid are low in plant protein products (53). Interestingly, tryptophan and glycine were somewhat more abundant in Egyptian children. While levels of tryptophan are higher in seeds and nuts, which are consumed more frequently in Mediterranean countries (53), this amino acid is not easily accessible from many products such as cereals (54). Elevated levels of fecal glycine were previously noted after dietary supplementation of fructo-oligosaccharides (55), which are found at high levels in fruits. Similarly to the majority of amino acids, we found that the levels of metabolites related to lipid metabolism, including bile acids, taurine (which can be derived through deconjugation of primary bile acids), and choline, were all higher in the American volunteers. Because release of bile acids into the small intestine is increased in subjects on a high-fat diet (56), more bile acids likely reach the colon in subjects consuming fat-rich diets, which aligns with our observations. Levels of several central metabolism and fermentation intermediates (ethanol, lactate, malate, pyruvate, afnd succinate) were also higher in U.S. samples, possibly indicating incomplete fermentation of complex polysaccharides in the guts of these teenagers (57). Intriguingly, the abundance of 1-methylhistamine, a fecal biomarker of allergic response (58), was also higher in uskHLT stools, consistent with the well-established higher prevalence of allergic diseases in industrialized countries (59).

10.1128/mSystems.00169-16.3 FIG S3  Mean H1 NMR spectral profiles from the distal guts of Egyptian and U.S. children. The averages of log_2_-transformed binned signal values within the egkHLT and uskHLT groups are visualized as line graphs. The positions of specific individual metabolites are indicated on the line graphs based on their respective chemical shifts (in parts per million). Download FIG S3, TIF file, 2.1 MB. Copyright © 2017 Shankar et al. 2017 Shankar et al. This content is distributed under the terms of the Creative Commons Attribution 4.0 International license.

10.1128/mSystems.00169-16.4 FIG S4  Comparison of distal gut metabolite profiles between Egyptian and U.S. groups using binned NMR data set. (A) Unconstrained PCA of centered log-ratio-transformed binned values show separation of distal gut metabolite profiles based on groups. (B and C) Results of discriminant analyses of visualizations of supervised random forest (RF) (B) and orthogonal projection to latent structures (OPLS-DA) (C) performed using a centered log-ratio-transformed binned data set. Statistical significance of group separation on plots is based on the Davies-Bouldin index. Group clouds represent areas of three standard errors around the group centroid (diamond). Download FIG S4, TIF file, 0.6 MB. Copyright © 2017 Shankar et al. 2017 Shankar et al. This content is distributed under the terms of the Creative Commons Attribution 4.0 International license.

10.1128/mSystems.00169-16.5 FIG S5  Bar graphs of Calinski-Harabasz index and Dirichlet multinomial mixture Laplace approximation values for *k* clusters. Calinski-Harabasz index values (A) calculated for each *k* value ranging from 2 to 20 and Laplace model approximations (model fit) from the Dirichlet multinomial mixtures (B) for *k* ranging from 2 to 11 are visualized as bar graphs. Higher CH index values and lower Laplace approximations indicate the more optimal clustering of the data set. Download FIG S5, TIF file, 0.2 MB. Copyright © 2017 Shankar et al. 2017 Shankar et al. This content is distributed under the terms of the Creative Commons Attribution 4.0 International license.

10.1128/mSystems.00169-16.8 TABLE S3  Fractional abundances of quantitated distal gut luminal metabolites from the Egyptian and U.S. children. Download TABLE S3, XLSX file, 0.04 MB. Copyright © 2017 Shankar et al. 2017 Shankar et al. This content is distributed under the terms of the Creative Commons Attribution 4.0 International license.

10.1128/mSystems.00169-16.9 TABLE S4  Gini scores and mean decrease in accuracy obtained for each random forest variable. Download TABLE S4, XLSX file, 0.01 MB. Copyright © 2017 Shankar et al. 2017 Shankar et al. This content is distributed under the terms of the Creative Commons Attribution 4.0 International license.

These differences in measured metabolite levels between studied cohorts were sufficiently consistent within each group to distinguish the egkHLT and uskHLT samples. Exploratory PCA, as well as discriminant RF analysis, separated Egyptian and U.S. samples in the ordination space based on the relative abundances of 32 measured metabolites (Fig. 3C and D). Coinertia analysis (CIA) indicated that such sample separation was congruent with the dispersion of the same samples in the genus-based ordination analysis (Fig. 3E).

Utilizing the SparCC algorithm to account for the limitations of compositional data (28), we also generated networks of intermetabolite correlations shared among all samples (Fig. 3B). Not surprisingly, levels of SCFAs, nucleotide metabolites, and amino acids all correlated strongly within each group. An overall negative correlation was observed between levels of SCFAs and amino acids (excluding tryptophan and glycine), consistent with nutritional differences between Mediterranean and Western diets.

### Abundances of gut microbial functions are consistent with the prevalent Egyptian and U.S. diets.

**FIG 4:**
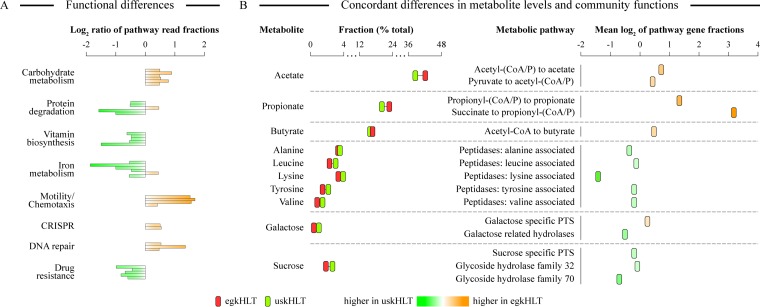
Functional analysis of the distal gut microbiota metagenomes. (A) Differences in specific functional categories between Egyptian and U.S. gut microbiota. Each category comprises several SEED level 3 entries, each represented as individual bars with results calculated as log_2_-transformed ratios of reads between egkHLT and uskHLT metagenomes. All displayed entries are statistically significant, with FDR-corrected *P* of <0.01. (B) Relative abundances of specific metabolites and the mean log_2_ ratios of reads of metabolic enzymes that produce and/or utilize the metabolites are compared. CoA/P, coenzyme A/phosphate.

To establish a link between microbial composition in the gut and the levels of luminal metabolites, we carried out functional metagenomic profiling of both sets of stool samples. Statistically significant differences in the abundances of many functional genes were observed between the egkHLT and uskHLT cohorts (the full table of functional annotations is provided in Data Set S1 in the supplemental material). Specifically, concordant with the high consumption of cereals by Egyptians, many carbohydrate utilization pathways were more abundant in the egkHLT stools (see Fig. 4A). These pathways included cellulosome complex; catabolism of d-galactarate, d-glucarate, d-glycerate, and d-gluconate; mannitol and melibiose utilization; and enzymes of the pentose phosphate pathway. In contrast, many protein degradation pathways, including general protein degradation modules, general aminopeptidases, and degradation enzymes for specific amino acids such as lysine and histidine, were more abundant in the gut microbiota of U.S. children. Interestingly, and in concordance with our metabolite quantification, tryptophan catabolism-related genes were more prevalent in the Egyptian fecal samples. Biosynthetic pathways for several vitamins, including biotin, cobalamin, and vitamin K, were significantly more abundant in the guts of the U.S. children (average ratio, 1.68), likely because consumption of many refined products such as sugars, cereals, and vegetable oils, which are low in micronutrients and vitamins, leads to lower vitamin consumption (60). Several iron acquisition systems were also more prevalent in the guts of U.S. teenagers (see Fig. 4A and Data Set S1). It was shown previously that the intestinal barrier function in Egyptian children is occasionally compromised and thus that more iron can leak from mucosal tissues into intestinal lumen (61). At the same time, U.S. teenagers underconsume foods rich in this metal (60).

10.1128/mSystems.00169-16.10 DATA SET S1  Functional annotation and statistical analyses of metagenomics reads. Download DATA SET S1, XLSX file, 0.6 MB. Copyright © 2017 Shankar et al. 2017 Shankar et al. This content is distributed under the terms of the Creative Commons Attribution 4.0 International license.

The overall abundance of motility and chemotaxis operons and quorum-sensing genes was higher in the Egyptian samples; this is consistent with the observed differential abundances of members of phylum *Proteobacteria*, which contains many known human gut pathogens (3.3% and 2.5% weighted mean relative abundances among egkHLT and uskHLT samples, respectively). Clustered regularly interspaced short palindromic repeat (CRISPR), restriction-modification, and DNA repair systems were also more abundant in the bacterial genomes in egkHLT samples (Fig. 4A), indicating that the Egyptian gut microbiota is under heavier bacteriophage pressure (62, 63). In contrast, levels of genes in the category of “resistance to antibiotics and toxic compounds” (including resistance to vancomycin and β-lactam antibiotics) were significantly higher in U.S. samples, a finding which is likely explained by the widespread use of antibiotic treatments in the United States, resulting in selective advantages of microbial genome-carried antibiotic resistance genes.

To further validate the observed links between the fecal microbiota and metabolites, we also linked the differences in individual fatty acid, sugar, and amino acid levels between egkHLT and uskHLT cohorts to the levels of enzymes producing or utilizing these metabolites (see Fig. 4B). The abundances of all SCFA-producing fermentation pathway enzymes were higher in the Egyptian samples, in agreement with higher SCFA abundances in these samples. Interestingly, the abundances of sucrose- and galactose-specific glucoside hydrolase enzymes as well as of sucrose- and fructose-specific phosphotransferase systems (PTS) were higher in the uskHLT samples, matching the higher fecal sucrose and galactose metabolite levels. Peptidases specific to the release of alanine, leucine, lysine, tyrosine, and valine from proteins and peptides were also more prevalent in the U.S. gut microbiota, concordant with the higher amino acid levels in uskHLT stools (Fig. 4B).

## DISCUSSION

The human physiological state is currently viewed as an interaction between a person’s genotype and the environment. This might explain in part why gene variants identified in many recent genome-wide studies can account for only a small proportion of the heritability of most complex diseases (64). Similarly, a recent alarming increase in the rates of obesity and excessive weight cannot be attributed to any genetic changes occurring on such short evolutionary time frame, and environmental factors such as diet, levels of physical activity, and compromised immune systems all contribute substantially to this obesity epidemic (65). The historic rate of dietary changes during the development of human species far exceeded that of possible genotype alterations (2). Thus, our genotype is presumably not well adapted to the current abundance of refined grains and sugars, dairy products, and animal fats in the diets of industrialized populations (1, 4, 66). These considerations are used to explain the observations that a Mediterranean diet, which is rich in plant products and low in animal fats, is associated with lower risk of cardiovascular diseases (11). The human gut microbiota serves as an important bridge connecting diet to human metabolism, since emerging evidence points to the metabolic mediation by the microbiota of both harmful and beneficial effects of dietary nutrients on human health (67, 68).

In this study, we explored the microbial and metabolic differences in the gut environment between two groups of adolescents—Egyptians consuming a Mediterranean diet and U.S. teenagers fed a typical Western diet. Integrative analysis of microbiota composition and functional capacity coupled with quantitative measurements of intestinal metabolites provided strong matching evidence of the differences between these populations. It appears that the gut microbiota in each population has adapted, at least in part, to the host’s prevalent diet. Thus, the Egyptian gut microbiota was enriched in polysaccharide-degrading members and genome-encoded enzymatic functions, whereas microbial communities in U.S. teenagers had higher counts of protein-degrading microbes and were enriched in protein and fat utilization pathways as well as in biosynthesis of the vitamins that are often found at low levels in Western diets. Microbiota adaptation to each gut environment was also evident from the overabundance of iron scavenging genes in the gut of U.S. children, consistent with the reports of insufficient iron consumption in subjects on a Western diet (60). Such differences in microbiota structure and function were reflected in the differences in the intestinal metabolites. While the gut environment of Egyptian teenagers was characterized by an abundance of short-chain fatty acids, intestines of U.S. children had increased amino acid content, higher levels of lipid metabolism-associated compounds, and elevated concentrations of 1-methylhistamine, a biomarker for allergic reactions. SCFAs, especially butyrate, inhibit inflammation and protect against obesity (69, 70), whereas products of protein and lipid degradation are associated with a risk of developing atherosclerosis and colon cancer (67, 71). Thus, the observed differences in these metabolites between our cohorts are consistent with the epidemiological data showing higher rates of cardiovascular disease, metabolic syndrome, colorectal cancer, and autoimmune and allergic diseases in industrialized populations (72).

While it is tempting to assume that gut microbiota transformation of dietary nutrients plays a central role in the development of these diseases, other environmental, cultural, and genetic contributions, as well as study limitations, should also be taken into account. Factors such as health care, hygiene practices, cultural variation, and environmental exposures to toxins and pathogens are all likely to exert selection pressures on the gut microbiome and metabolome. For example, while the diet appears to be “healthier” in the Egyptian population, the rate of obesity is actually higher in Egypt among the members of the adult population, especially women, than in the United States. This seeming inconsistency can be potentially explained by several factors: the recent proliferation of “junk food” outlets in Egyptian cities (all our Egyptian teenagers were provided prepared meals and thus were not exposed to these sources of foods); increasingly sedentary lifestyles; and the lack of opportunities to play sports and to exercise (73). In this study, only male preadolescent and adolescent subjects were recruited; the gut environment in female teenagers was not profiled. Children in many developing countries, including Egypt, also often suffer from the environmental enteric dysfunction that can lead to nutrient malabsorption, altered immunity, and changes in the gut microbiota. In many cases, this enteric dysfunction might be caused by altered barrier function and reduced absorptive surface of the intestinal epithelium, and it is often associated with poorer hygiene and a higher prevalence of pathogens in the environment (74). Finally, acquisition of more-detailed dietary data in future studies should provide additional insights into associations of specific microbes and metabolites with particular types of foods. Nevertheless, because microbes can evolve and adapt to environmental changes much more rapidly than humans can, dietary modifications are likely to be among the most efficient and, at the same time, low-cost options for prevention and treatment of metabolic and immune diseases (75). Thus, modulation of human gut microbiota with prebiotic, probiotic, and synbiotic dietary supplementations, or through microbiota transplantation, can provide new approaches to control the diet-microbiota-human health interactions in the near future.

## MATERIALS AND METHODS

### Study subjects.

Fresh fecal samples were collected in sterile containers from healthy preadolescent and adolescent male volunteers from Giza, Egypt (designated “egkHLT”; *n* = 28, average age = 13.9 ± 0.6 years; average body mass index [BMI] = 18.9 ± 2.5 kg/m^2^), and from Dayton, OH (designated “uskHLT”; *n* = 14, average age = 12.9 ± 2.8 years; average BMI = 21.2 ± 3.4 kg/m^2^). Both cohorts were living in the urban setting. Fresh fecal samples were homogenized immediately after collection and were frozen within 0 to 2 h after defecation as described previously (76, 77). The subject enrollment was limited to males to take advantage of the availability of a teenage male cohort in a welfare institution in Cairo, Egypt. Healthy volunteers did not have any gastrointestinal symptoms and had not consumed antibiotics or probiotics for at least 3 months prior to sample collection. For each volunteer, age and BMI values were collected and used in data interpretation.

### Isolation of gDNA and high-throughput DNA sequencing.

Total genomic DNA (gDNA) was isolated from 150 mg of fecal material using a ZR fecal DNA isolation kit (Zymo Research Corporation) according to manufacturer’s protocol. For the interrogation of microbial composition, the V4 variable region of the 16S ribosomal RNA gene was amplified using the universal primers 515F (5′-GTGCCAGCMGCCGCGGTAA) and 806R (5′-GGACTACHVGGGTWTCTAAT). The forward primers contained an 8-nucleotide barcode to permit sample pooling. PCR amplifications were performed in a 25-μl volume with 25 ng of genomic DNA and 28 cycles of amplification. PCR products were cleaned and purified using calibrated AMPure XP beads (Beckman Coulter, Inc.). Amplicons were equimolarly pooled and processed using the Illumina TruSeq DNA library preparation protocol. Sequencing was performed on an Illumina MiSeq platform using the 2× 250-nucleotide-paired-end sequencing protocol following the manufacturer’s guidelines. An average of 72,039 ± 34,259 reads were obtained per sample. Paired-end sequence reads were joined together. Low-quality (average *Q*, <25) and short (<150-bp) reads were removed from the data set. High-quality reads were analyzed in QIIME using the default pipeline (78). Operational taxonomic units (OTUs) were defined by clustering at 97% sequence similarity. Taxonomic annotation of OTUs was performed with the UCLUST algorithm (79) against curated GreenGenes database v13.8 (80). All OTU counts were adjusted to calculated taxon 16S rRNA gene copy numbers using the rrnDB library (81) in order to represent true relative abundances (76, 77). Finally, the adjusted reads from all samples were subsampled at the read level (rarefied) such that all samples were represented by the same number of counts. This final data set was used for all multivariate analyses.

To analyze community functional capacity, shotgun metagenomic sequencing was employed. Whole-community genomic DNAs from individual samples were equimolarly pooled within each group. Each pooled DNA sample was fragmented and processed using the Illumina TruSeq DNA library preparation protocol. Sequencing was performed on an Illumina MiSeq platform using the 2× 150-bp-paired-end sequencing protocol. Totals of 17,655,028 and 14,534,774 reads were obtained for egkHLT and uskHLT samples, respectively. All reads were uploaded into the MG-RAST analysis server (82). Paired reads were combined and subjected to quality filtering, and host sequences were depleted. The data set was then processed using the default MG-RAST analysis pipeline. The functional annotation was based on the SEED hierarchical system (83). STAMP statistical software was used for visualization and statistical hypothesis testing based on the two-sample Fisher exact test with *P* values adjusted for multiple-hypothesis testing using the Benjamini-Hochberg false-discovery-rate (FDR) algorithm (84). The following filters were used for the selection of level 3 entries from the SEED hierarchical annotation for in-depth analysis: (i) entries with greater than 500 reads; (ii) entries with a value for fold change between sample groups of at least 1.3; (iii) entries with consistent changes among functional genes within the same group.

In order to determine the abundances of genes encoding carbohydrate active enzymes, the protein sequences of all available glycoside hydrolases (GH), glycosyl transferases (GT), polysaccharide lyases (PL), and carbohydrate esterases (CE) were downloaded from the CAZy database (85). A BLASTP search was then used to match metagenomic sequence reads against this custom annotation database. Individual families clustered in a manner depending on the enzyme class substrate specificity and function. A similar approach was used to annotate gene reads encoding galactose- and-sucrose specific phosphotransferase systems (PTS).

### Fluorescent *in situ* hybridization (FISH).

FISH was carried out based on the methods of Zhu and Joerger (86). *Bacteroides* and *Prevotella* were visualized using newly designed fluorescein isothiocyanate (FITC)-labeled probes Bfra602 (5′-GAGCCGCAAACTTTCACAA) and Prev743 (5′-AATCCTGTTCGATACCCGCA). The probes were designed to be specific to each genus with no cross-hybridizing to any other genera. The ability of each probe to detect members of the corresponding genus was checked via the probe match function of the Ribosomal Database Project’s 16S rRNA gene database, and the correct hybridization was validated using pure cultures of *Bacteroides fragilis* and *Prevotella oralis*. To carry out FISH, bacterial cells were isolated from 100 mg of fecal material with phosphate-buffered saline (PBS)–0.1% SDS buffer and were fixed overnight with a 4% paraformaldehyde–PBS solution. Fixed cells were treated with lysozyme and then hybridized with the appropriate fluorescent probe at 46°C for 16 h using a probe-specific hybridization solution (86, 87). Fluorescent images of DAPI (4′,6-diamidino-2-phenylindole)-stained and FITC-stained cells were captured through a 100× oil immersion objective using Image-Pro 6.2 software. Eight fields were imaged per hybridization. Total cell counts were obtained from images of DAPI-stained cells, and the ratio of probe-hybridized cells to total cells was determined using FITC images.

### Preparation of fecal water extracts and proton nuclear magnetic resonance (NMR) analysis.

A total of 250 mg of homogenized stool was used to prepare a metabolite water extract in phosphate buffer following our previously described protocol (57). A 550-µl aliquot of the prepared fecal extract sample was transferred to a 5-mm-inner-diameter NMR tube together with 150 µl of 9 mM trimethylsilylpropionic-2,2,3,3-d_4_ acid (TSP) in D_2_O. Proton (^1^H) NMR spectra were acquired at 25°C using a Varian Inova instrument operating at 600 MHz (14.1 Tesla) and a previously described procedure (57). TSP served as a chemical shift reference and quantification standard, and D_2_O provided a field-frequency lock for NMR acquisition. Data were signal averaged over 400 transients using a 4.0-s acquisition time and an interpulse delay of 11.05 s. Spectral processing included removal of the residual water signal, chemical shift referencing, and sum normalization. For multivariate data analyses, spectra were binned to reduce the dimensionality and mitigate peak misalignment, and signal intensities were autoscaled (88). Quantification of specific metabolite resonances was accomplished using an interactive spectral deconvolution algorithm in MatLab as previously described (57). The deconvolution tool fits a defined spectral region using a combination of tunable baseline shapes (spline, v-shaped, linear, or constant) and a Gauss-Lorentz peak-fitting function. All metabolite peak intensities were corrected for equivalent numbers of protons and normalized to the TSP signal intensity. Peak metabolite assignments were either taken from our previous study (57) or confirmed in additional metabolite spike-in experiments. In the latter cases, each metabolite compound was added as a spike-in into a baseline fecal extract at a final concentration of 1.5 mM, the NMR spectrum was acquired, and the position of the metabolite-identifying peak was confirmed. There was substantial variability in the total sum of 32 metabolites among samples; the variability was not a function of sample group or sample water content. To increase the robustness of downstream analyses, all metabolite values were converted into fractions of the total.

### Statistical data analyses.

Statistical procedures were carried out in R, SPSS v19 (SPSS, Inc.), and MatLab (the MathWorks, Inc.). Weighted mean values around the median were calculated to obtain sample group averages as previously described (77). Weighted mean values reduce the effect of outliers on the mean estimate. The statistical significance of observed differences in the values of any quantitative variables between sample groups was assessed by the Mann-Whitney *U* test (reported as *p*_*U*_ values after FDR adjustment) (84). Beta diversity was measured with ecological Bray-Curtis and phylogenetic UniFrac distances (25, 89). Multivariate ordination analyses were carried out to assess sample dispersal as a function of microbial and metabolite profiles. Prior to the analyses, genus and phylotype relative abundance data sets were subjected to chord transformation to account for many zero values, and the metabolite abundance data set was subjected to centered log-ratio transformation to correct for data compositionality (28). Principal-component analysis (PCA), principal-coordinate analysis (PCoA), Unifrac distance-based redundancy analysis (db-RDA), db-RDA-based variation partitioning, random forest (RF) analysis, and orthogonal projections to latent structures discriminant analysis (OPLS-DA) were run in MatLab and R. A Venn diagram of variation partitioning was constructed with eulerAPE (90). The statistical significance of group separation in PCA and PCoA was tested using the permutation of the Davies-Bouldin index measure (91). Performance of discrimination models was assessed based on the out-of-bag error rates. Identification of microbial enterotypes within the chord-transformed genus abundance data set was achieved by partitioning around the medoid (PAM) analysis (48) and between-cluster analysis (BCA) in R as previously described (46). The Calinski-Harabasz (CH) index was calculated for different numbers of clusters (between 2 and 20 clusters) to determine the number of clusters that provided the optimal sample distribution. The separation of samples into two clusters provided the best CH index value. This finding was further validated by calculating Silhouette scores in R.

To test the sample distribution congruency between the microbiota and metabolite data sets, transformed genus and metabolite relative abundance data sets were subjected to coinertia analysis (CIA) (28). CIA was performed in R, and permutation of the RV coefficient was used to test the significance of the congruency. To assess putative associations among the quantified metabolites in all samples, the metabolite fractional abundance data set was analyzed in SparCC (92). The statistical significance of observed correlations was calculated through comparison to null distributions generated by permutation and renormalization of data.

### Data accessibility.

Sequence data sets from 16S ribosomal RNA gene sequencing and metagenomic sequencing supporting the conclusions of this article are available in the Sequence Read Archive Repository (BioProject identifier [ID] PRJNA314988) and the MG-RAST analysis server (accession IDs: for egkHLT, 4552772.3 [http://metagenomics.anl.gov/linkin.cgi?metagenome=mgm4552772.3] and 4552773.3 [http://metagenomics.anl.gov/linkin.cgi?metagenome=mgm4552773.3]; for uskHLT, 4552774.3 [http://metagenomics.anl.gov/linkin.cgi?metagenome=mgm4552774.3] and 4552775.3 [http://metagenomics.anl.gov/linkin.cgi?metagenome=mgm4552775.3]).
