# Supplementary figures and images for: Differences in Gut Metabolites and Microbial Composition and Functions between Egyptian and U.S. Children Are Consistent with Their Diets
Source: mSystems. 2017 Feb 7;2(1):e00169-16. doi: 10.1128/mSystems.00169-16 (PMC5296411; doi:10.1128/mSystems.00169-16)

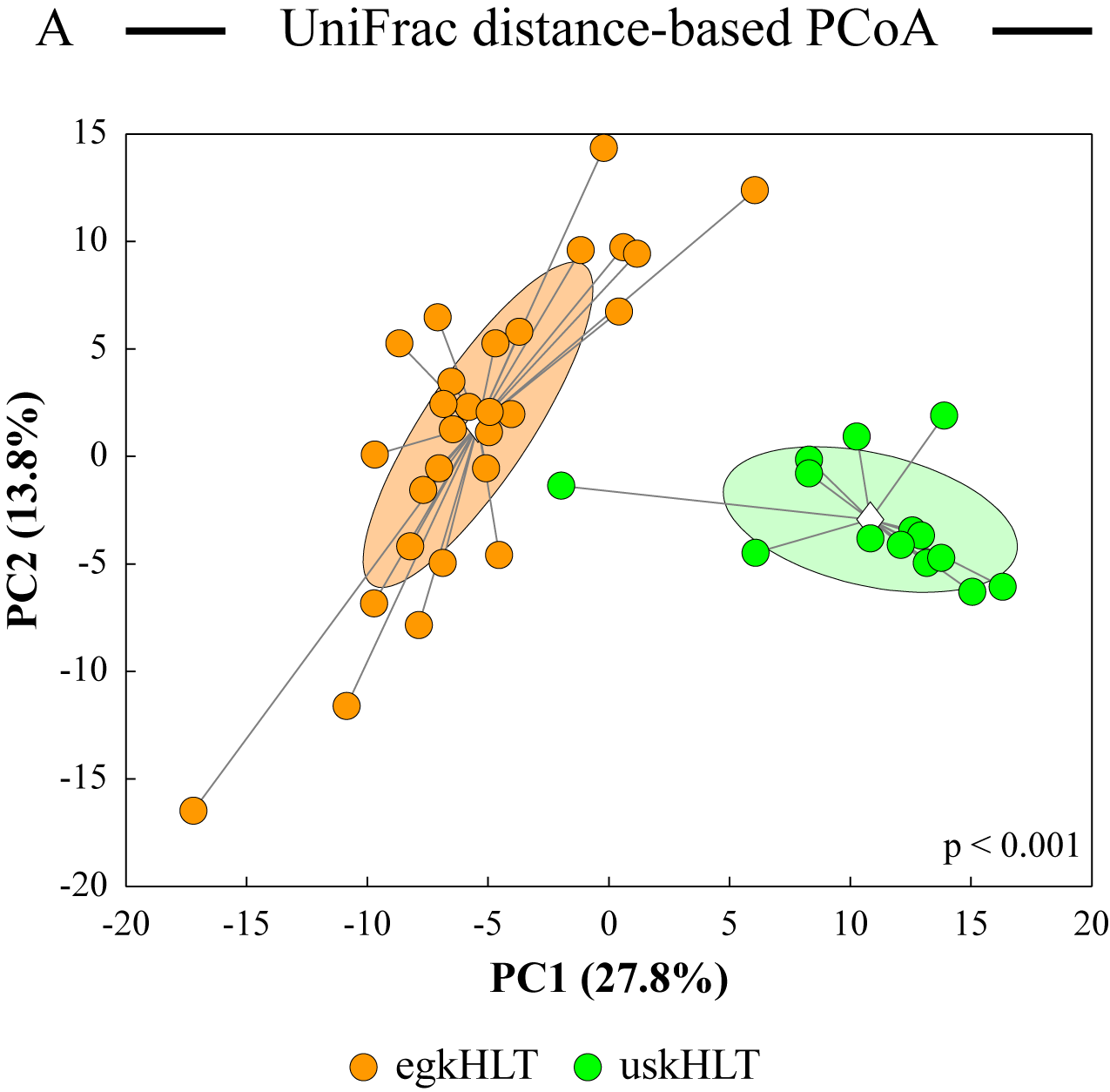

Supplement: FIG S1 [file sys001172084sf1.tif]

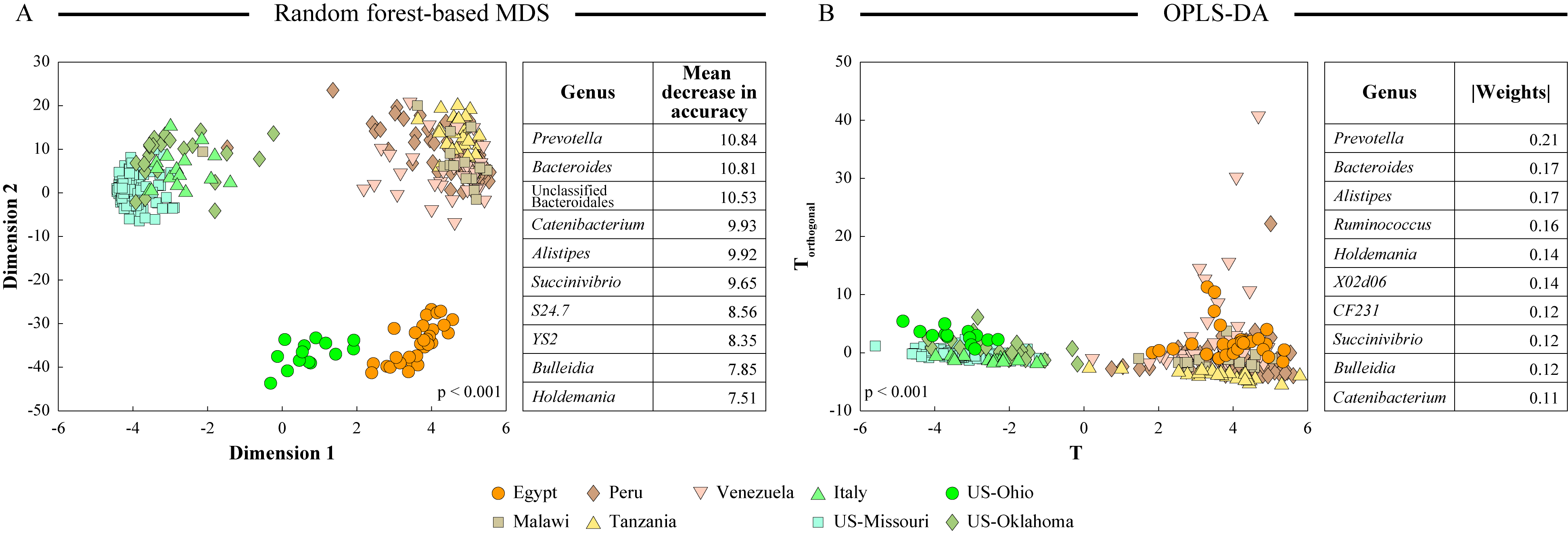

Supplement: FIG S2 [file sys001172084sf2.tif]

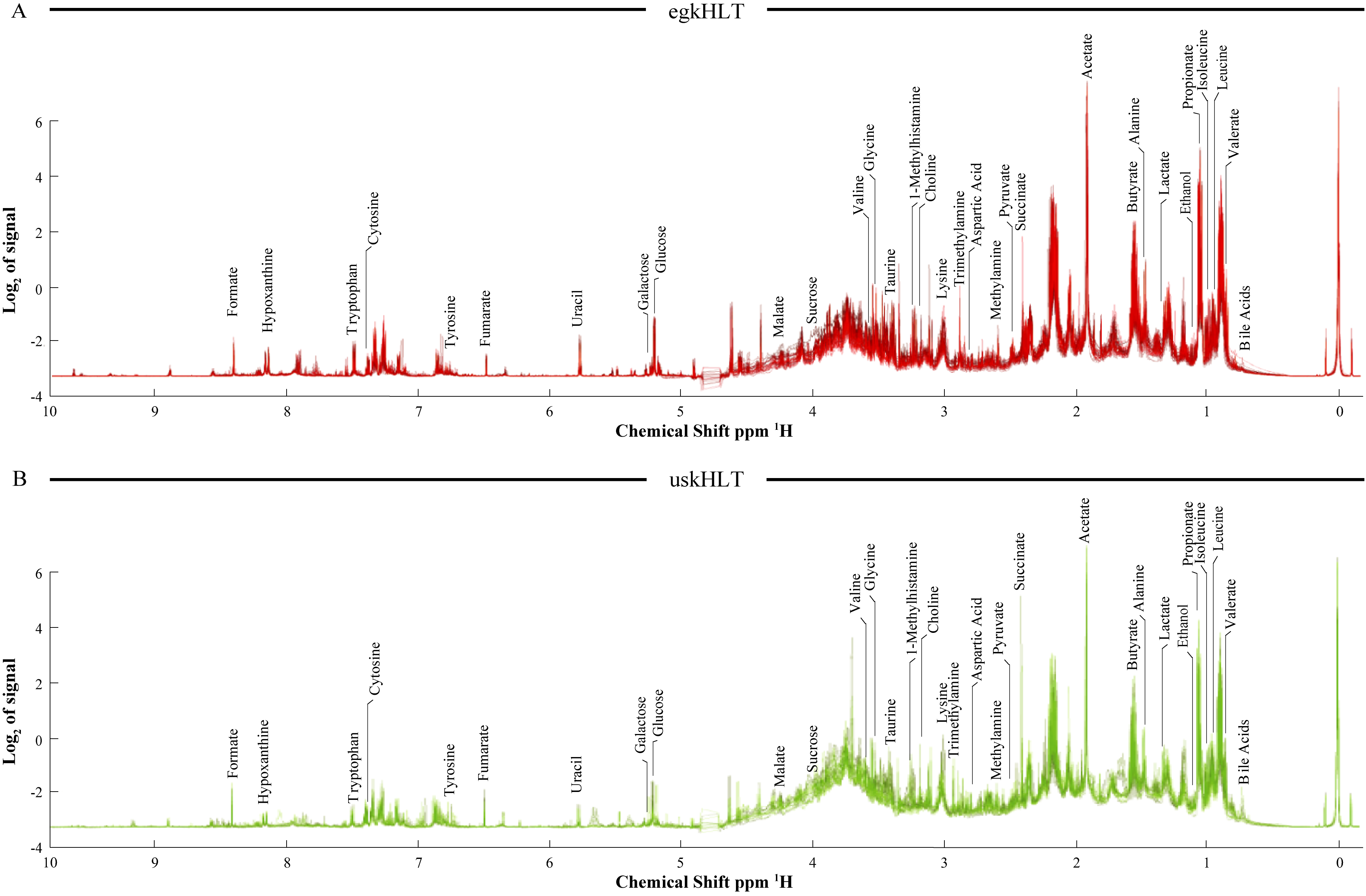

Supplement: FIG S3 [file sys001172084sf3.tif]

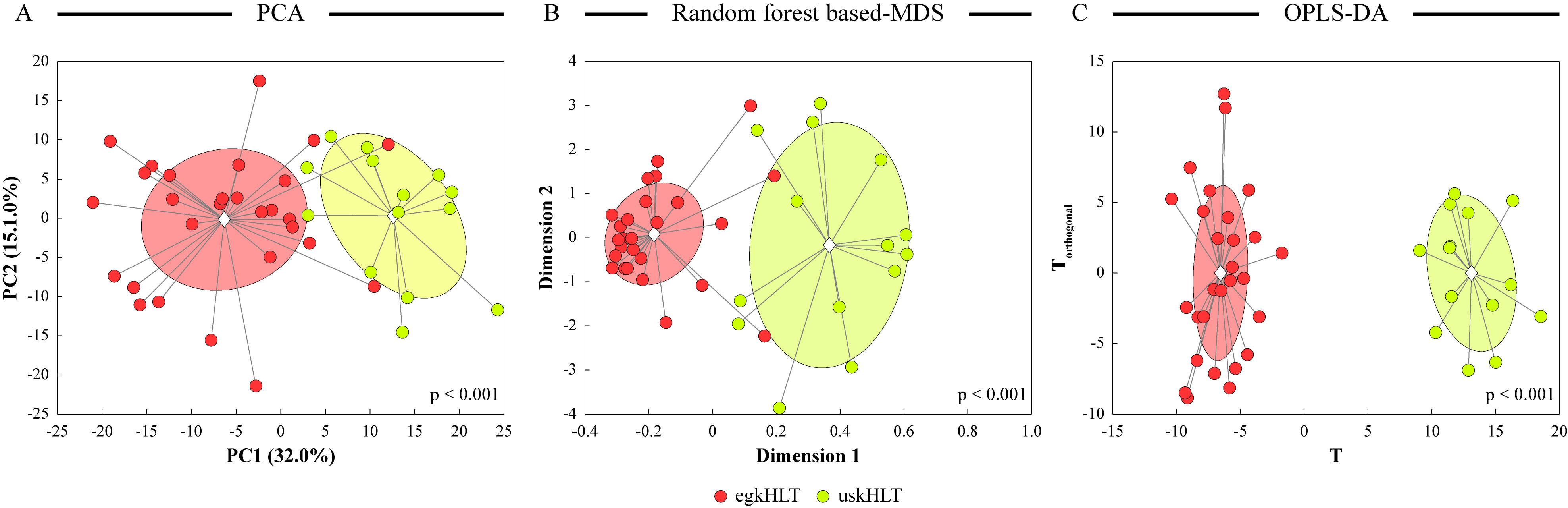

Supplement: FIG S4 [file sys001172084sf4.tif]

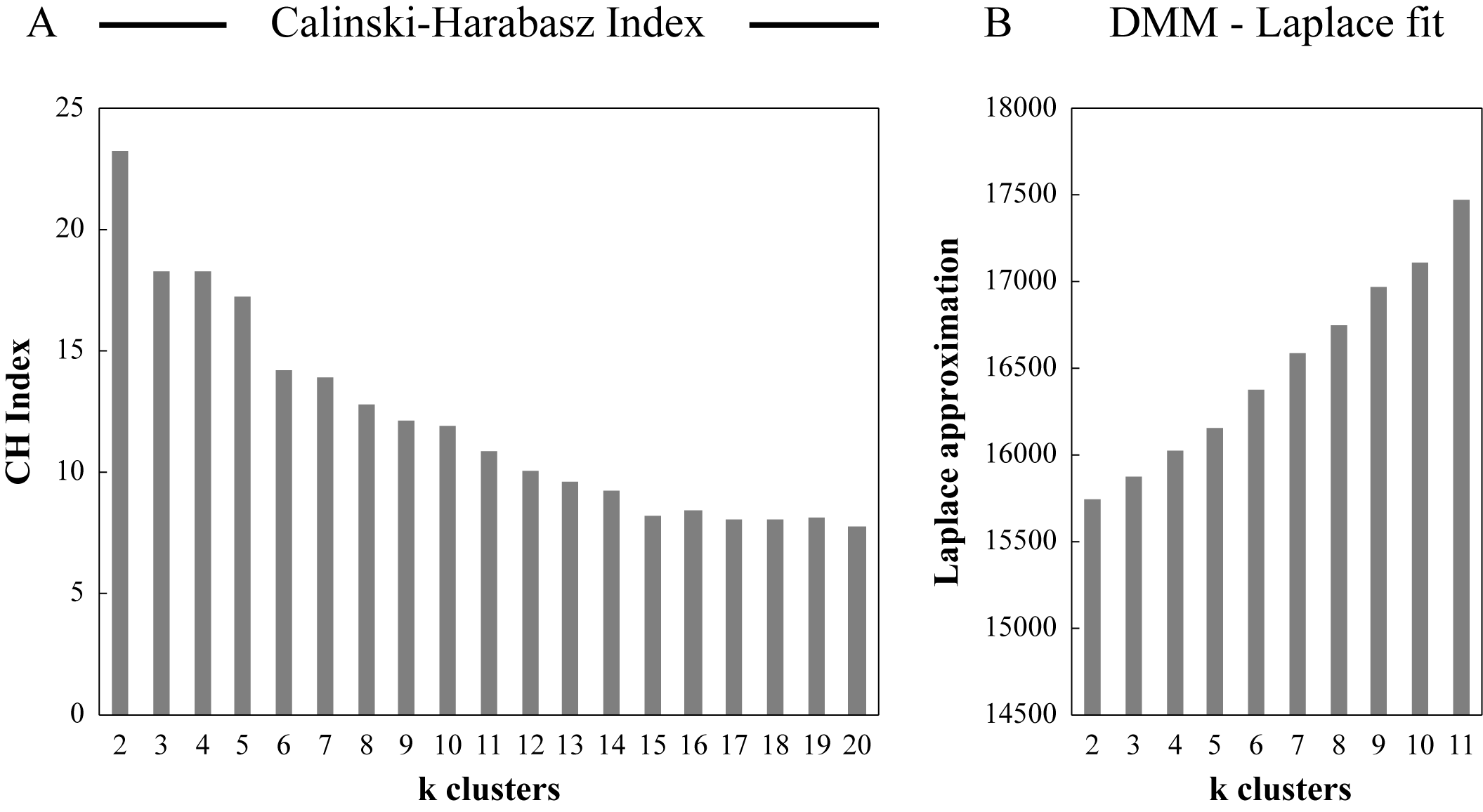

Supplement: FIG S5 [file sys001172084sf5.tif]
